# Distributed Collaborative Data Processing Framework for Unmanned Platforms Based on Federated Edge Intelligence

**DOI:** 10.3390/s25154752

**Published:** 2025-08-01

**Authors:** Siyang Liu, Nanliang Shan, Xianqiang Bao, Xinghua Xu

**Affiliations:** 1 National Key Laboratory of Electromagnetic Energy, Naval University of Engineering, Wuhan 430033, China; m24181105@nue.edu.cn (S.L.); nanliang@stu.xmu.edu.cn (N.S.); xinghuaxv@nue.edu.cn (X.X.); 2 East Lake Laboratory, Wuhan 430202, China

**Keywords:** unmanned platforms, federated learning, edge computing, data sharing, hierarchical parameter alignment, similarity coefficient, loss gradient

## Abstract

Unmanned platforms such as unmanned aerial vehicles, unmanned ground vehicles, and autonomous underwater vehicles often face challenges of data, device, and model heterogeneity when performing collaborative data processing tasks. Existing research does not simultaneously address issues from these three aspects. To address this issue, this study designs an unmanned platform cluster architecture inspired by the cloud-edge-end model. This architecture integrates federated learning for privacy protection, leverages the advantages of distributed model training, and utilizes edge computing’s near-source data processing capabilities. Additionally, this paper proposes a federated edge intelligence method (DSIA-FEI), which comprises two key components. Based on traditional federated learning, a data sharing mechanism is introduced, in which data is extracted from edge-side platforms and placed into a data sharing platform to form a public dataset. At the beginning of model training, random sampling is conducted from the public dataset and distributed to each unmanned platform, so as to mitigate the impact of data distribution heterogeneity and class imbalance during collaborative data processing in unmanned platforms. Moreover, an intelligent model aggregation strategy based on similarity measurement and loss gradient is developed. This strategy maps heterogeneous model parameters to a unified space via hierarchical parameter alignment, and evaluates the similarity between local and global models of edge devices in real-time, along with the loss gradient, to select the optimal model for global aggregation, reducing the influence of device and model heterogeneity on cooperative learning of unmanned platform swarms. This study carried out extensive validation on multiple datasets, and the experimental results showed that the accuracy of the DSIA-FEI proposed in this paper reaches 0.91, 0.91, 0.88, and 0.87 on the FEMNIST, FEAIR, EuroSAT, and RSSCN7 datasets, respectively, which is more than 10% higher than the baseline method. In addition, the number of communication rounds is reduced by more than 40%, which is better than the existing mainstream methods, and the effectiveness of the proposed method is verified.

## 1. Introduction

With the rapid development of artificial intelligence and Internet of Things technology, unmanned platforms such as drones and unmanned aerial vehicles have shown great potential in the fields of environmental monitoring, disaster relief, logistics, and distribution [1]. However, with the increase in task complexity, a single unmanned platform has difficulty meeting the needs of large-scale and multi-scenario cooperative operations [2], Yan et al. proposed a collaborative confrontation decision-making method for heterogeneous drone swarms, based on self-play reinforcement learning, which enhanced the intensity of drone confrontation and further optimized maneuvering strategies [3]. Shi et al. verified the autonomous collaborative capabilities of drone swarms in a meta-battlefield domain, demonstrating their situational awareness, intelligent response, and autonomous formation capabilities under complex environments and limited resources [4]. Xue et al. introduced a distributed task assignment algorithm based on coalition formation game theory, which achieved effective task allocation while significantly improving real-time performance and maximizing the efficiency of the swarm [5]. Liu et al. incorporated a quantum decision-making model to address the autonomous decision-making problem in heterogeneous swarms, enhancing the applicability and transferability of drone swarms [6]. However, these studies focus on the level of mission decision-making, and the solution of how to carry out cooperative data processing on unmanned platforms is still insufficient.

In the scenario of cooperative task execution by unmanned platforms, the cooperative data processing capability of unmanned clusters plays a vital role in ensuring the efficient and accurate completion of tasks [7]. Indu et al. proposed a multi-hop data off-loading scheme which proved the feasibility of decentralized data processing and off-loading in UAV networks [8]. Wang et al. used UAV time series data and recurrent neural networks to accurately predict yield at the field scale, providing a basis for timely and precise agricultural decision-making [9]. Wei Min et al. proposed a federal privacy-preserving UAV data collection framework, which improves the route accuracy of UAV path planning, strengthens data security, and reduces carbon emissions by 20% [10]. However, its strict data isolation mechanism exacerbates the problem of data fragmentation (non-independent and identically distributed, Non-IID) in the dynamic networking scenarios of unmanned platforms, significantly reducing the model convergence speed. The above-mentioned schemes for collaborative data processing in unmanned platforms struggle to simultaneously meet the requirements of privacy security, heterogeneity compatibility, computational efficiency, etc.

The Federated Edge Intelligence (FEI) method provides a new idea for cooperative unmanned platform data processing by integrating the privacy protection characteristics of federated learning and the near-source processing ability of edge computing. This technology aims to achieve efficient model training and knowledge sharing in a distributed environment, while reducing communication overhead. It is a frontier research direction in the field of unmanned platform collaborative computing.

Federated learning [11], a distributed machine learning approach, leverages the computational power of different nodes. It enables distributed data storage and processing to accelerate model training and improve performance. After local data training, participants share and aggregate model parameters, not raw data, achieving knowledge sharing and model optimization while ensuring data privacy and security. In an unmanned cluster environment, federated learning supports unmanned platforms to co-train a shared model while protecting data privacy, which can directly transfer model parameters between unmanned platforms instead of raw data, and it can be used to improve the efficiency of data sharing, which reduces the burden of communication and improves the efficiency of intelligent data processing. Federated learning can also resist malicious attacks and protect the security of the model, which plays a key role in the stable operation of unmanned swarms in complex environments.

Existing research on federated learning has primarily focused on addressing the issues of heterogeneity and privacy protection in federated learning. For instance, Sun et al. developed a personalized federated multitask learning algorithm with knowledge distillation, improving each client’s personalized accuracy under heterogeneous models [12]. Zhang et al. proposed a federated class-incremental learning method combining hybrid knowledge distillation in a digital twin environment, enhancing performance of federated class-incremental models in Non-IID data conditions [13]. Liu et al. introduced an adaptive encoder allocation model for better device heterogeneity solutions [14]. Yin et al. proposed a federated local momentum acceleration learning algorithm with attention mechanisms to alleviate data heterogeneity [15]. These algorithms mostly focus on single-dimension heterogeneity optimization, struggling to cope with the complex scenarios where data, device, and model heterogeneities interweave in unmanned platforms. Xiong presented a federated learning algorithm based on device clustering and differential privacy, which effectively enhanced model accuracy and convergence speed [16]. Rodríguez et al. constructed a classification system for adversarial attack and defense methods in federated learning, and proposed a guide for selecting defense methods, thereby providing a comprehensive framework for research on the security of federated learning [17]. Ballhausen et al. proposed the “federated secure computing” architecture, which separates cryptography from business logic through a simple API, supports multiple privacy-preserving computing protocols, and verified its efficiency on Internet of Things (IoT) devices, thus lowering the threshold for the application of privacy-preserving computing [18]. Karras et al. proposed a client-balanced Dirichlet sampling algorithm with probabilistic guarantees to alleviate the problem of oversampling, optimize the data distribution among clients, and thereby achieve more accurate and reliable model training [19].

In the practical application of federated learning for unmanned platforms, it also faces high latency and bandwidth pressure caused by data transmission, making it difficult to meet the strict real-time requirements of complex tasks. Edge computing [20] reduces latency and bandwidth consumption and improves computational efficiency by off-loading computing tasks to an unmanned platform closer to the data source. By collaboratively completing all computing tasks on edge nodes, edge computing not only optimizes task offloading scheduling, but also takes into account the communication cost, real-time computing capacity, and residual energy of each node; thus, the scheduling efficiency and endurance of the unmanned swarm are effectively improved.

**Figure 1:**
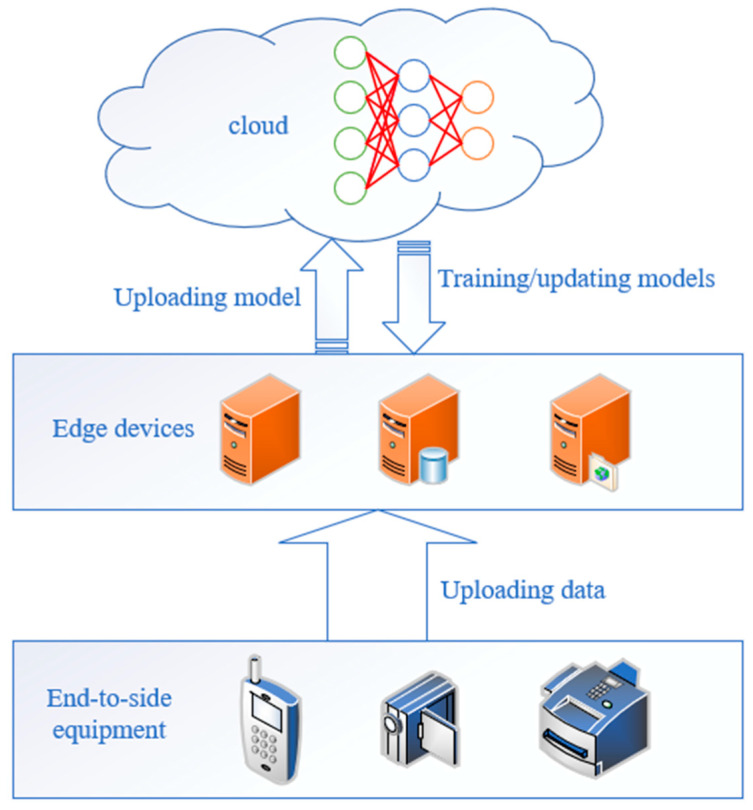
Schematic of cloud-edge-end architecture.

The Cloud-Edge-End architecture [21], as a novel distributed computing paradigm, enables the construction of a highly collaborative and intelligent unmanned swarm system. As illustrated in Figure 1, the cloud, based on in-depth data analysis, promptly pushes trained or updated models to the edge and end devices, formulating macro-level strategies and plans for the entire system. The edge, leveraging its proximity to the data source, quickly receives data collected by end devices and utilizes its onboard computational resources to run relatively simple intelligent algorithms and models. The end devices, as the components most directly interfacing with the physical world or users, collect raw data using various sensors and transmit the collected data to the edge or cloud in a timely and accurate manner.

**Figure 2:**
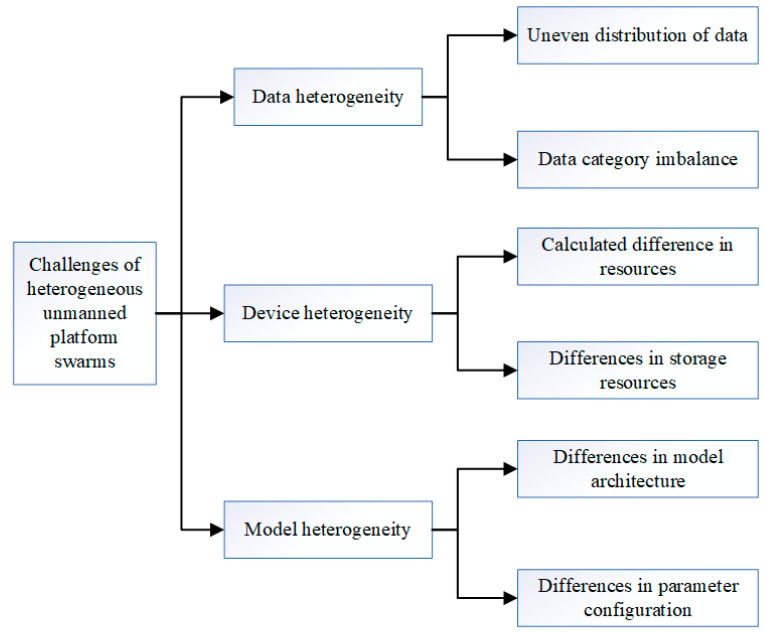
Challenges faced by heterogeneous unmanned platform swarms.

As shown in Figure 2, the main challenges for collaborative data processing on unmanned platforms in a cloud-edge-end architecture are as follows:

(1) Data heterogeneity [22]: During the process of data collection and processing by large-scale unmanned platforms, due to varying task requirements and environmental conditions, different platforms may gather data with significantly different categories. Additionally, the numbers of samples collected by each platform may be unevenly distributed [23]. These factors result in the data collected by unmanned platforms exhibiting non-independent and identically distributed (Non-IID) characteristics.
(2) Device heterogeneity: Unmanned platforms vary significantly in terms of computational resources, storage resources, and other aspects. This heterogeneity necessitates that algorithms and models in collaborative operations must adapt to the hardware limitations of different devices to achieve optimal resource allocation and utilization.
(3) Model heterogeneity [24]: Different unmanned platforms may face diverse task requirements, necessitating the use of varying model architectures or parameter configurations.
(4) Privacy protection [25]: Traditional data processing methods typically involve transmitting collected data to a centralized data processing center (such as a cloud server) for storage and analysis. During data transmission, due to the openness of the network, data is susceptible to interception by third parties. In this process, data privacy faces significant risks.

The cooperative data processing ability of unmanned platform directly affects its task execution efficiency in a dynamic environment. The existing methods often lead to slow convergence and insufficient generalization ability due to data heterogeneity and equipment differences. In view of the above problems, this paper proposes a federated edge intelligence method (DSIA-FEI) that integrates data sharing and intelligent model aggregation strategies, which significantly improves the accuracy, convergence speed, and stability of unmanned swarms for collaborative data processing. The main contributions and innovations of this paper are as follows:

(1) This paper proposes a federated edge computing architecture for unmanned platform clusters, integrating technologies such as federated learning and edge computing into the cloud-edge-end paradigm. Compared with traditional federated learning methods, this architecture better adapts to the dynamic and distributed application scenarios of unmanned platforms through three-level collaboration: global scheduling at the cloud layer, distributed processing at the edge layer, and data collection at the terminal layer.
(2) In the federated edge intelligence framework, to address the issues of data class imbalance and large distribution differences among edge platforms, a privacy-enhanced data sharing mechanism is introduced. While mitigating the problem of data distribution heterogeneity across platforms, this mechanism strengthens data privacy protection by adding random perturbations to data using Gaussian noise.
(3) This paper proposes an intelligent model screening strategy that combines similarity measurement and loss gradient. The strategy first uses a hierarchical parameter alignment method to map the parameters of heterogeneous models to a unified space; then, according to the similarity coefficient and loss gradient, the local model that is most beneficial to the global model aggregation is selected, which significantly reduces the interference of device and model heterogeneity on the global model aggregation in the federated learning process.

## 2. Cloud-Edge-End Architecture for Unmanned Platform Swarms

The study, based on the cloud-edge-end collaborative computing paradigm, designs a three-layer federated edge computing architecture for unmanned swarms. This architecture effectively integrates the distributed model training advantages of federated learning with the near-source data processing capabilities of edge computing. While ensuring data privacy and security, it significantly enhances the overall task execution efficiency of the swarm. The specific details of the federated edge intelligence method within this architecture are provided in Section 3.

**Figure 3:**
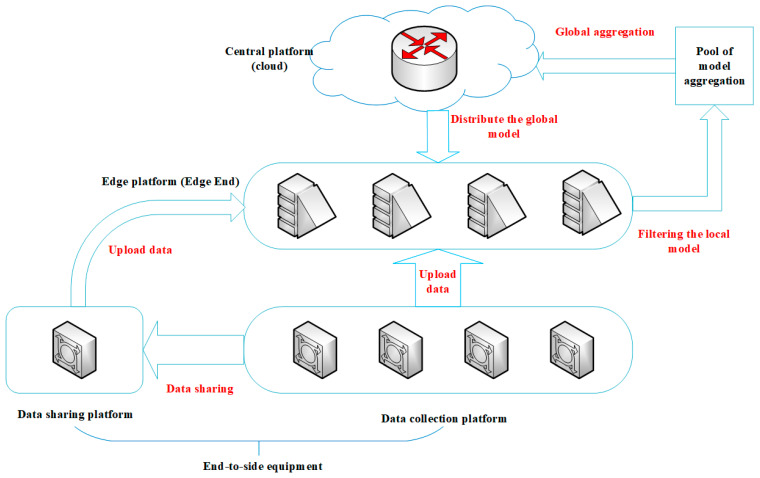
Swarm architecture of unmanned platform.

As shown in Figure 3, the proposed architecture consists of three layers: the central platform, edge platforms, and terminal platforms, forming an efficient and collaborative intelligent computing network. Specifically, on the terminal side, sensor-equipped platform nodes are responsible for environmental perception and data collection, as well as performing necessary data preprocessing operations. On the edge side, deployed edge platforms act as computational relay nodes, not only receiving and processing data transmitted from the terminal layer but also conducting local model training and parameter optimization, enabling near-source data processing. In the cloud layer, the central platform serves as the global coordinator, responsible for aggregating model parameters from various edge nodes and managing the updates and distribution of the global model.

This hierarchical architecture design, through the introduction of a federated edge intelligence framework, achieves multiple advantages:

(1) At the data level, it ensures the privacy and security of raw data while facilitating cross-platform knowledge sharing.
(2) At the computational level, localized processing at edge nodes significantly reduces data transmission overhead.
(3) At the model level, a hierarchical collaborative training mechanism is employed, ensuring both the timeliness and accuracy of model updates.

## 3. Method of Federated Edge Intelligence

In the distributed model training process of unmanned platform swarms, edge platforms face two primary challenges when training models based on local datasets: First, due to the non-independent and identically distributed (Non-IID) nature of the data [11], there exists a significant discrepancy between local data distribution and the global distribution. Second, the heterogeneity in computational capabilities and model architectures further exacerbates the divergence between local models and the global model. These factors severely impact the convergence speed and final performance of the model.

Existing federated learning methods largely rely on parameter averaging for aggregation, which is based on the implicit assumption of independent and identically distributed (Non-IID) data. This assumption is easily violated in the context of heterogeneous unmanned platform sensor data and diverse task scenarios, leading to model bias. To compensate for this bias, regularization or local fine-tuning is often required, which significantly slows down the convergence speed. Moreover, for edge nodes equipped with heterogeneous models, global aggregation becomes challenging under such circumstances.

**Figure 4:**
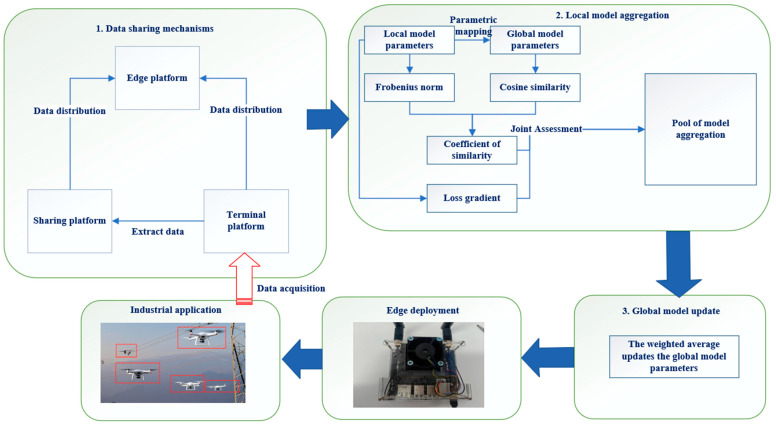
Schematic diagram of federated edge intelligent methods.

To address these challenges, this paper proposes an innovative federated edge intelligence method—data sharing and intelligent model aggregation strategy Federated Edge Intelligence (DSIA-FEI), which integrates data sharing and model similarity measurement. As shown in Figure 4, this method introduces a dynamic data-sharing mechanism, which effectively mitigates the negative impact of non-independent and identically distributed (Non-IID) data. Meanwhile, it performs hierarchical parameter alignment between local and global models and employs a similarity loss gradient-based model selection strategy. This reduces the interference of device and model heterogeneity on global model aggregation, thereby significantly improving the efficiency of distributed training and model performance.

### 3.1. Data Sharing Strategy

Federated learning relies on local model updates uploaded by each participant to aggregate global models. When the data distribution is heterogeneous, the model will perform well on some participants’ data and poorly on other participants’ data. Because the global model is based on the aggregation of local models, the distribution difference will lead to the model not being able to fully adapt to the data characteristics of all participants. In the process of edge cooperative data acquisition and transmission of unmanned platform clusters, there are often cases of unbalanced data categories and uneven distribution, leading to slow convergence speed, model performance degradation, communication efficiency reduction, and other issues in the subsequent model training. In order to alleviate the problem of heterogeneous data distribution, this paper introduces a data sharing mechanism. The edge collaboration process after using the data sharing mechanism is as follows:

(1) Data Sampling: In the terminal platforms, data is sampled according to a predefined sharing ratio. Research indicates that model accuracy plateaus when the data-sharing ratio is between 20% and 30% [11]. Beyond this range, further increasing the sharing ratio does not lead to unlimited accuracy gains. Thus, in the subsequent experiments, the sharing ratio was set at 20%. Additionally, data normalization and noise reduction were performed to eliminate redundant components from the original data.
(2) Data Transmission: The sampled data is transmitted to the data-sharing platform. This process necessitates ensuring the accuracy and efficiency of data transfer.
(3) Data Integration: The data-sharing platform integrates the received data to form a public dataset. This process involves data cleaning and preprocessing to ensure data quality.
(4) Data Distribution: The common data in the data sharing platform is randomly sampled and distributed to each edge platform. The edge platforms utilize both the public dataset and their local datasets for model training.

By sharing the public dataset, the data discrepancies among different edge platform are reduced. This enhancement contributes to the stability and reliability of the entire unmanned platform swarm, improving the central platform’s ability to generalize local models from various edge platforms and the local models’ capability to generalize datasets.

When applying data sharing strategies in unmanned platforms, to implement privacy protection, sensitive information (such as UAV IDs, geographical locations, etc.) can be deleted, only retaining features required for model training. Controllable noise is injected into the data, where Gaussian noise adds random perturbations to continuous data. Its probability density function is as follows:

(1) $$f\left(x\right)=\frac{1}{\sqrt{2\pi \sigma^{2}}}e^{-\frac{{\left(x-\mu \right)}^{2}}{2\sigma^{2}}}$$

Specifically, the standard deviation σ serves to control the noise amplitude, which enables the blurring of sensitive information while preserving the data distribution characteristics. However, the noise amplitude needs to be quantitatively regulated according to the signal-to-noise ratio (SNR); the calculation method of SNR is as follows:

(2) $$SNR\left(dB\right)=10\log_{10}\frac{P_{signal}}{P_{noise}}$$

where SNR denotes the magnitude of the signal-to-noise ratio, $P_{signal}$ represents the power of the original data, and $P_{noise}$ stands for the power of the Gaussian noise.

### 3.2. Intelligent Model Aggregation Mechanism

Under the federated learning framework, the significant divergence between local models on edge platforms and the global model introduces adverse effects: Firstly, the participation of low-quality local models in global aggregation degrades the overall performance of the model, compromising the data processing accuracy of the unmanned platform swarm. Secondly, transmitting these inefficient models consumes valuable communication resources, exacerbating the load pressure on resource-constrained platforms [26]. This issue is particularly pronounced in scenarios involving non-independent and identically distributed (Non-IID) data.

From the perspective of model representation, the heterogeneity in local data distributions is directly reflected in the dynamic variations of the model’s weight matrix. The weight matrix exhibits dual characteristics:

- Numerical Characteristics: The absolute values of the matrix elements reflect the strength of neuronal connections, indicating the model’s focus on different features.
- Directional Characteristics: Treating the weight matrix as a vector in high-dimensional space, its directional information implicitly captures the learning trends and convergence direction of the model, analogous to semantic direction indicators in vector space models.

To ensure the effectiveness of model aggregation, accurately measuring the similarity between local models and the global model is crucial. However, traditional similarity metrics face limitations in simultaneously capturing both numerical differences and directional characteristics when dealing with high-dimensional neural network weight matrices. This paper proposes a dynamically evolving model similarity calculation method based on the Frobenius norm and cosine similarity. This approach uses the Frobenius norm [27] to quantify numerical differences in the weight matrix and leverages cosine similarity to capture the directional properties of the model, thereby achieving a comprehensive evaluation of model similarity. A weight parameter α is introduced to balance the influence of these two metrics on model similarity.

The similarity coefficient reflects the consistency and correlation between local and global models in the feature space, while the loss gradient of a model captures the update dynamics during training, indicating the direction and magnitude of parameter changes. Significant changes in a local model’s loss function suggest that new, valuable feature patterns have been learned from the local environment.

Accordingly, this paper advances an intelligent model aggregation strategy that combines similarity coefficients and loss gradients. This approach aims to identify local models that contribute positively to global model updates.

#### 3.2.1. Alignment of Hierarchical Parameters

When calculating the similarity coefficient between the global model and the local model or performing global model aggregation, it is necessary to ensure that the model parameter structure matches. However, in the federated edge intelligence scenario for unmanned platform swarms, each unmanned platform node may deploy heterogeneous local models (such as CNN, Resnet, etc.) due to task requirements and resource constraints. Although the overall architecture of the model is different, some layers (such as convolutional layers, fully connected layers) assume similar functions in feature extraction or classification tasks. Therefore, this paper proposes a method of hierarchical parameter alignment, which can be used to improve the accuracy of feature extraction and classification, mapping heterogeneous parameters to a unified space by hierarchical parameter alignment.

The global model of a terminal platform contains K functional layers $L=\{l_{1},l_{2},\ldots ,l_{K}\}$, and each layer $l_{K}$ is defined as a class of functional modules, such as a convolution layer, residual layer, attention layer, etc. Similarly, for the local model $M_{i}$ in the i-th edge platform, its local layer is aligned with the global layer according to its functional similarity.

For the local layer L with similar functions in the i-th edge platform, its parameter dimensions may not be the same, so that it is necessary to map the parametric dimension $\theta_{k}^{\left(i\right)}\in R^{d_{k}^{\left(i\right)}}$ of L into the global-level unified parameter space $\theta_{k}\in R^{d_{k}}$ by the parametric mapping function $\phi_{k}^{i}:R^{d_{k}^{\left(i\right)}}⟶R^{d_{k}}$.

For the convolution layer, let the local convolution kernel size be $h^{i}\times w^{i}$ and the global convolution layer size be h×w; then the mapping can be performed by bilinear interpolation, as shown in (3).

(3) $${\tilde{\theta }}_{k}^{i}=Resize\left(\theta_{k}^{i},\;h\times w\right)$$

where, for the target location (x,y) in the convolution layer, the four adjacent points are $\left(x_{1},y_{1}\right)$, $\left(x_{1},y_{2}\right)$, $\left(x_{2},y_{1}\right)$, $\left(x_{2},y_{2}\right)$, and the bilinear interpolation formula is shown in Formula (4).

(4) $$\tilde{\theta_{k}}\left(x,y\right)=\sum_{i=1}^{2}\sum_{j=1}^{2}\theta_{k}\left(x_{i},y_{j}\right)\times \left(1-\left|x-x_{i}\right|\right)\times \left(1-\left|y-y_{j}\right|\right)$$

For the fully connected layer, let the dimension of the local layer weight matrix be $m^{i}\times n^{i}$, the dimension of the global layer weight matrix be m×n, and the mapping be performed by zero-padding or truncation, as shown in Formula (5).

(5) $$\tilde{\theta_{k}}\left(p,q\right)=\left\{\begin{array}{cc} \theta_{k}^{i}\left(p,q\right), & \mathrm{if}\;p\leq \;m^{i},q\leq n^{i}\\ 0, & \mathrm{otherwise} \end{array}\right.$$

In the residual layer, the structure of the module includes the convolution of the main path and the jump connection. The parameter mapping needs to align the main path parameters and process the jump connection according to the global model structure. For each convolution layer $W_{cov}$ of the main path and the size of the jump connection, Formula (3) is also used to align the size of the convolution kernel and the number of channels in the primary path, and the skip connection is aligned using a 1 × 1 convolution:

(6) $$\left\{\begin{array}{c} \tilde{W_{cov}}=W_{1\times 1}\times W_{cov}\\ \tilde{W_{shortcut}}=W_{1\times 1}\times W_{shortcut} \end{array}\right.$$

It should be noted that the number of channels is a structural parameter of the model rather than a weight parameter, and will not participate in the subsequent similarity measurement and model aggregation. The number of channels is mapped to keep the model structure consistent so that effective global aggregation can occur.

After parameter mapping, in order to eliminate the difference in the dimension of different local model parameters, the mapped weight parameters are normalized, as shown in Formula (7)–(9).

(7) $${\tilde{\theta }}_{k}^{i}=\frac{{\tilde{\theta }}_{k}^{i}-\mu_{k}}{\sigma_{k}}$$

(8) $$\mu_{k}=E\left(\tilde{\theta_{k}}\right)$$

(9) $$\sigma_{k}=\sqrt{E{\left(\tilde{\theta_{k}}-\mu_{k}\right)}^{2}}$$

#### 3.2.2. Coefficient of Similarity and Loss Gradient

The Frobenius norm [28,29] can directly quantify the numerical difference between the corresponding elements of two matrices. For the weight matrix, calculating the Frobenius norm of the weight matrix of the global model and the local model can clearly quantify the degree of deviation between the models. The smaller the Frobenius norm is, the higher the similarity between models is. For the global model weight matrix G and the local model weight matrix $L_{i}$, the Frobenius norm is calculated as shown in Formula (10).

(10) $${\left\|\left.G-L_{i}\right\|\right.}_{F}=\frac{1}{k}\gamma_{i}\sum_{i=1}^{k}\sqrt{\sum_{m=1}^{M}\sum_{n=1}^{N}{\left|\left.{\left(G-L_{i}\right)}_{ij}\right|\right.}^{2}}$$

where $\gamma_{i}$ has a value of 0 or 1: 0 indicates that the functional module does not exist in the local model of the edge platform; otherwise, it is 1.

The Frobenius norm only focuses on the numerical difference of the numerical weight matrix, and ignores the consistency or difference information of the weight matrix as a vector set in the direction, which cannot reflect whether the model learning trend is synergistic. The cosine similarity can capture the direction relationship between vectors, and the cosine similarity can be calculated after the weight matrix is expanded into a vector form by row or column, which can reveal the similarity of the model in the direction of feature learning. The higher the cosine similarity of the two weight matrices, the closer their directions in the high-dimensional space, that is, the model’s extraction patterns of data features are similar. After the global model weight matrix G and the local model weight matrix $L_{i}$ are flattened into vectors, the cosine similarity calculation method is as shown in Formula (11).

(11) $$\;cos<G,L_{i}>=\frac{1}{k}\gamma_{i}\sum_{i=1}^{k}\frac{\sum_{i=1}^{m\times n}GL_{i}}{\sqrt{\sum_{i=1}^{m\times n}G^{2}}\sqrt{\sum_{i=1}^{m\times n}L_{i}^{2}}}$$

After calculating the Frobenius norm and cosine similarity of the local and global models, the similarity coefficient of the local and global models is shown in Formula (12).

(12) $$\;S_{i}=\alpha cos<G,L_{i}>+\left(1-\alpha \right)\frac{1}{{\left\|\left.G-L_{i}\right\|\right.}_{F}}$$

In Formula (12), $S_{i}$ represents the similarity coefficient between the local model of the i-th edge platform and the global model. A higher value of $S_{i}$ indicates a greater degree of similarity between the models.

In federated learning, the loss gradient reflects the direction and speed of the model’s descent with the current parameters. In the early stage of model training, when the loss gradient is large, there is a large gap between the current parameters of the model and the optimal parameters, and the model needs to quickly adjust the parameters to reduce the loss function value. At this time, the cosine similarity can better measure the directional consistency between the local model and the global model, determine the reasonable update direction, and improve the accuracy of the local model, avoiding the deviation of the update direction from the optimal path caused by excessive attention to the numerical difference of parameters.

As the training proceeds, when the model enters the later fine-tuning stage, the learning direction of each node has become stable, and the loss function trend is stable, so that the model gradually focuses on the Frobenius norm. As the loss function is stable, at this time, the focus of optimization is to ensure the numerical accuracy of weight update, so as to effectively control the numerical influence of local updates on the global model and improve the overall performance of the model.

Therefore, in this paper, the α value is set to be related to the rate of change of the loss function as the iteration of the model gets smaller, which enables the model to better dynamically adjust the similarity coefficient according to the current training situation and task requirements, and improve the performance of the model, as shown in Equation (13).

(13) $$\alpha_{t}=\alpha_{t-1}-\beta \frac{L_{t-1}-L_{t}}{L_{t-1}}$$

Here, t represents the current iteration count, T denotes the initially set maximum number of iterations, $L_{t-1}$ is the value of the loss function in the last iteration, $L_{t}$ is the value of the loss function in the current iteration, and β is used to control the adjustment step size based on the relative change of loss, i.e., $\frac{L_{t-1}-L_{t}}{L_{t-1}}$. An excessively large β is prone to induce oscillations in the α value, whereas an excessively small β tends to result in a slow convergence rate of the α value.

In subsequent experiments, this study sets the initial value $\alpha_{0}$ = 0.8 to ensure the consistency of update directions between the local model and global model in the early stage of model training. Since the relative change in loss, i.e., $\frac{L_{t-1}-L_{t}}{L_{t-1}}$, always lies within the interval [−1, 1], β is set to 0.05 to ensure that the adjustment step size $\beta \frac{L_{t-1}-L_{t}}{L_{t-1}}$ is always less than 10% of $\alpha_{t-1}$, thereby preventing weight oscillations.

After calculating the similarity coefficient, the computation of the loss gradient for the i-th local model is illustrated in Formula (14).

(14) $$\nabla L_{i}=\left\|\frac{\partial L_{i}\left(\theta \right)}{\partial \theta }\right\|$$

#### 3.2.3. Model Aggregation Strategy

After computing each local model’s similarity coefficient $S_{i}$ and loss gradient $\nabla L_{i}$, we present an intelligent model aggregation strategy. Introducing aggregation thresholds for the similarity coefficient and loss gradient ($th_{s},th_{l}$) and a model aggregation pool (Agg) enables smart filtering of local models for global aggregation. In each federated learning iteration, the system compares $S_{i}$ with $th_{s}$ and $\nabla L_{i}$ with $th_{l}$ for each edge platform’s local model. A local model is added to the aggregation pool if its $S_{i}$ and $\nabla L_{i}$ exceed their respective thresholds. This mechanism effectively filters out low-quality local models, enhancing the global model’s robustness, convergence stability, and generalization ability.

Studies indicate that the similarity coefficient between local and global models is around 1.0 when datasets have no noisy labels [30]. Consequently, in subsequent experiments, the thresholds are set at $th_{s}$ = 1.0 and $th_{l}$ = 0.1.

After filtering out the appropriate local model, the global model of each functional layer L, using a weighted average approach to global aggregation, is shown in Formula (15).

(15) $$\theta_{k}=\sum_{i=1}^{k}\frac{n_{i}}{n}\theta_{k}^{\left(i\right)}$$

### 3.3. Federated Learning Process

Based on the theoretical analysis above, the workflow of federated learning for heterogeneous unmanned platforms is outlined in Algorithm 1.
**Algorithm 1** Federated Learning Algorithm**Input**Maximum number of iterations T
**Output**Global model parameters $\theta_{k}$
1**Initiate** 
$\theta_{k}^{0}$
2**for** ${node}_{i}=1$ **to**  Node3  **for** k=1 **to** K4    
$\theta_{k}^{\left(i\right)}\leftarrow \theta_{k}^{0}$
5  **end**
6**end**7**for** t=1 **to** T8  **for** ${node}_{i}=1$ **to** Node9    **for** k=1 to K10      
$\theta_{t+1}^{i,k}⟵\theta_{t}^{i,k}-\alpha_{t}\nabla f\left(\theta_{t}\right)$
11      Parametric mapping12    **end**
13    Calculate the coefficient of similarity and gradient of loss14    **if** $S_{i}\geq th_{s}\;and\;\nabla L_{i}\geq th_{l}$ **then**15      Agg [ ].append($\theta_{t+1}^{i}$)16    **end**
17**end**18**if** Agg [ ] = none **then**19  Aggregation of all local models20**end**21**end**22**for** k=1 to K23  
$\theta_{k}⟵\sum_{i=1}^{k}\frac{n_{i}}{n}\theta_{k}^{\left(i\right)}$
24**end**

## 4. Experimental Results and Analysis

In this paper, the edge intelligent device is used as a resource-constrained unmanned platform, and a variety of datasets are simulated to verify the effectiveness of the proposed method.

### 4.1. Dataset

**Figure 5:**
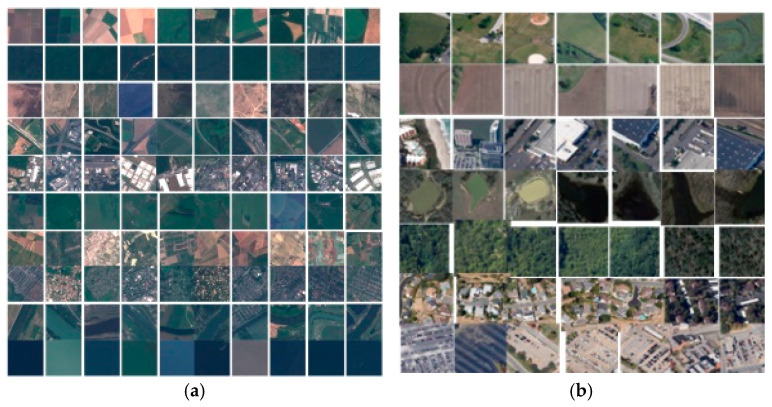
Sample graph of EuroSAT and RSSCN7 dataset. (**a**) EuroSAT; (**b**) RSSCN7.

This paper conducts experiments using the open-source FEMNIST, FLAIR, EuroSAT, and RSSCN7 datasets. FEMNIST is derived from the multi-writer EMNIST handwritten character dataset. There are differences in character categories and features across writers. FLAIR consists of around 430,000 images from 51,000 Flickr users. It has varying numbers of images per user and classes per collection. Images of the same class from different users exhibit distribution shifts. Both FEMNIST and FLAIR have inherent Non–IID properties [31]. EuroSAT contains a large number of high-resolution satellite images from various regions across Europe, covering 10 different land cover types such as farmland, forest, grassland, and rivers, with high spectral and spatial resolution. Meanwhile, RSSCN7 primarily focuses on remote sensing image data from China, encompassing seven categories including residential areas, woodland, grassland, water bodies, roads, bare land, and farmland. Examples of the EuroSAT and RSSCN7 datasets are illustrated in Figure 5.

**Figure 6:**
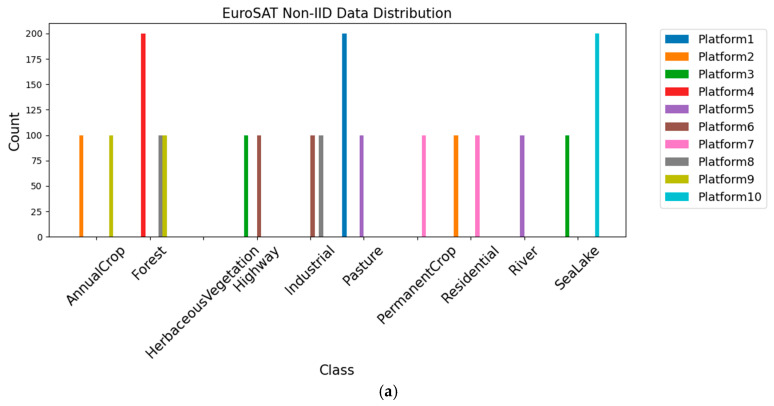

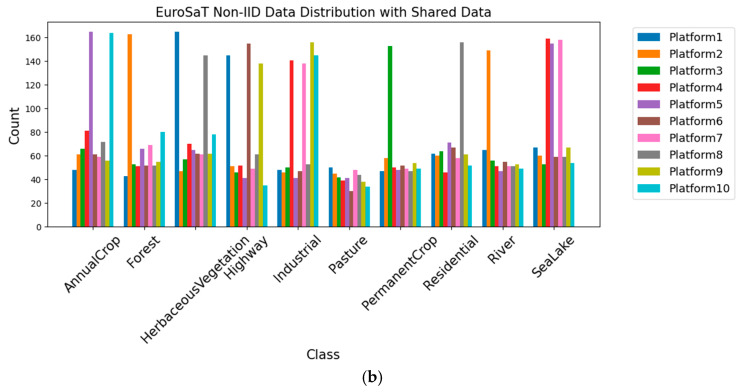
EuroSAT data distribution of each platform before and after data sharing strategy. (**a**) EuroSAT-Non-IID; (**b**) EuroSAT-Non-IID-shared.

**Figure 7:**
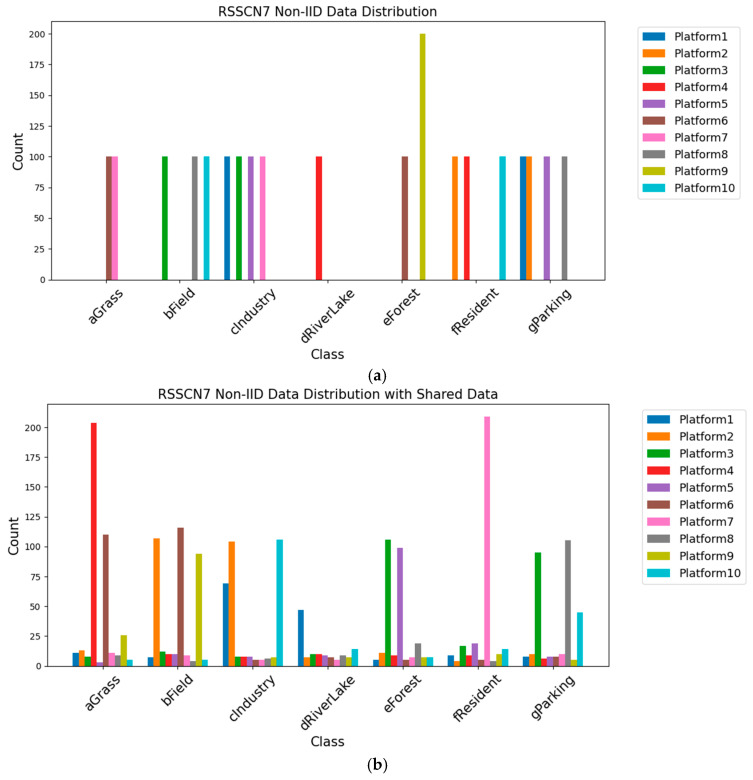
RSSCN7 data distribution of each platform before and after data sharing strategy. (**a**) RSSCN7-Non-IID; (**b**) RSSCN7-Non-IID-shared.

As illustrated in Figure 6 and Figure 7, in the subsequent experiments, the dataset is divided into multiple shards, which are distributed to individual edge platform in a Non-IID manner. Figure 6a and Figure 7a show the initial shard allocation for 10 platforms with Non-IID data. There are substantial disparities in data quantity across different classes on various platforms, reflecting a highly imbalanced and Non-IID data distribution. Figure 6b and Figure 7b depict the shard allocation after data sharing. While the Non-IID characteristics are still partially retained, the data distribution is improved with better and more balanced coverage of classes across platforms. This indicates that the data-sharing mechanism alleviates data distribution bias and class imbalance, providing a more solid foundation for federated learning model training.

### 4.2. Experimental Setup

#### 4.2.1. Assessment Indicators

To evaluate the effectiveness of the proposed federated edge intelligence method in enhancing the collaborative operations of unmanned platform swarms, three evaluation metrics were employed: precision, macro-averaged F1 score, and gradient divergence among different clients. Specifically, precision and macro-averaged F1 score were utilized to quantify the accuracy of the collaborative operations of the unmanned platform swarm, while gradient divergence was adopted to assess the consistency of model updates across different edge unmanned platforms.

(1) Accuracy: Accuracy reflects the model’s overall classification performance on all edge unmanned platforms’ data. However, when the dataset has Non-IID characteristics, accuracy may not indicate the model’s performance on minority classes, especially in cases of class imbalance.

(16) $$Accuracy=\frac{TP+TN}{TP+TN+FP+FN}$$

(2) F1_score: In Non-IID datasets, the number of samples for certain classes may be limited. The F1 score is better suited to reflect the model’s performance on minority classes. For a dataset with M classes, after calculating the F1 score for any individual class C (F1_score_C), the macro-averaged F1 score (macro_F1) is computed to evaluate the model’s classification capability across samples of different classes.

(17) $$Precision=\frac{TP}{TP+FP}$$

(18) $$Recall=\frac{TP}{TP+FN}$$

(19) $$F1\_score=2*\frac{Precision*Recall}{Precision+Recall}$$

(20) $$macro\_F1=\frac{1}{M}\;\sum_{C=1}^{M}F1\_score\_C$$

(3) ${Grad}_{diff}$: In Non-IID datasets, due to the differing data distributions across various edge platforms, gradient divergence is typically more pronounced. The magnitude of gradient divergence can effectively reflect the efficacy of the data-sharing strategy.

(21) $$\;{Grad}_{diff}=\sqrt{\sum_{i=1}^{N-1}{\left(\nabla f_{i}\left(\omega_{t}\right)-\nabla f_{i+1}\left(\omega_{t}\right)\right)}^{2}}$$

Here, TP,TN,FP,FN denote the counts of true positives, true negatives, false positives, and false negatives, respectively; $y_{ij}$ represents the predicted probability for the *i*-th sample; and $\nabla f_{i}\left(\omega_{t}\right)$ signifies the gradient value of the local model for the *i*-th edge platform.

(4) Rounds: Communication rounds are a key metric in federated learning that reflect how often participants exchange information during model training. They indicate how frequently client devices communicate with the central server for parameter updates and synchronization. Lower communication rounds mean less communication resource consumption. In this study, the number of communication rounds needed for the federated learning model to converge is used as an evaluation metric. A lower value of this metric indicates that the model consumes fewer communication resources.

#### 4.2.2. Baseline Methods

This paper employs five classical federated learning algorithms—FedAvg [11], FedProx [32], FedCosA [33], FedBN [34], and FedMAE [35]—as baseline methods. FedAvg is a classic federated learning algorithm that involves multiple rounds of iterative training. It trains models in parallel on multiple local clients and uploads local model parameters to the server for weighted averaging to update the global model. FedProx introduces a regularization term into the local objective function to constrain the difference between the local and global models, thereby alleviating the issue of data heterogeneity. FedCosA addresses the slow convergence, overfitting, and high communication costs in federated learning by integrating the Adam optimizer, a cosine annealing learning rate scheduler, and weight decay; it is especially suitable for Non-IID data scenarios. FedBN uses local batch normalization to alleviate the feature transfer prior to averaging model. FedMAE pre-trains a block’s shaded autoencoder (MAE) using large images in a lightweight client device, then cascades multiple pre-trained single-block Maes in a server, and finally generates a single block’s shaded autoencoder (MAE), to build a multi-block VIT backbone network for downstream tasks.

This paper integrates the hierarchical parameter alignment method into all baseline algorithms to handle heterogeneous models. It employs the FedAvg algorithm as the primary baseline method. Subsequently, ablation experiments are conducted to analyze the impact of incorporating data-sharing strategies and similarity-based aggregation strategies into the model. Finally, the proposed method in this paper is compared with the FedAvg, FedProx, FedCosA, FedBN and FedMAE algorithms through comparative experiments to validate the effectiveness of the proposed method.

#### 4.2.3. Training Settings

**Figure 8:**
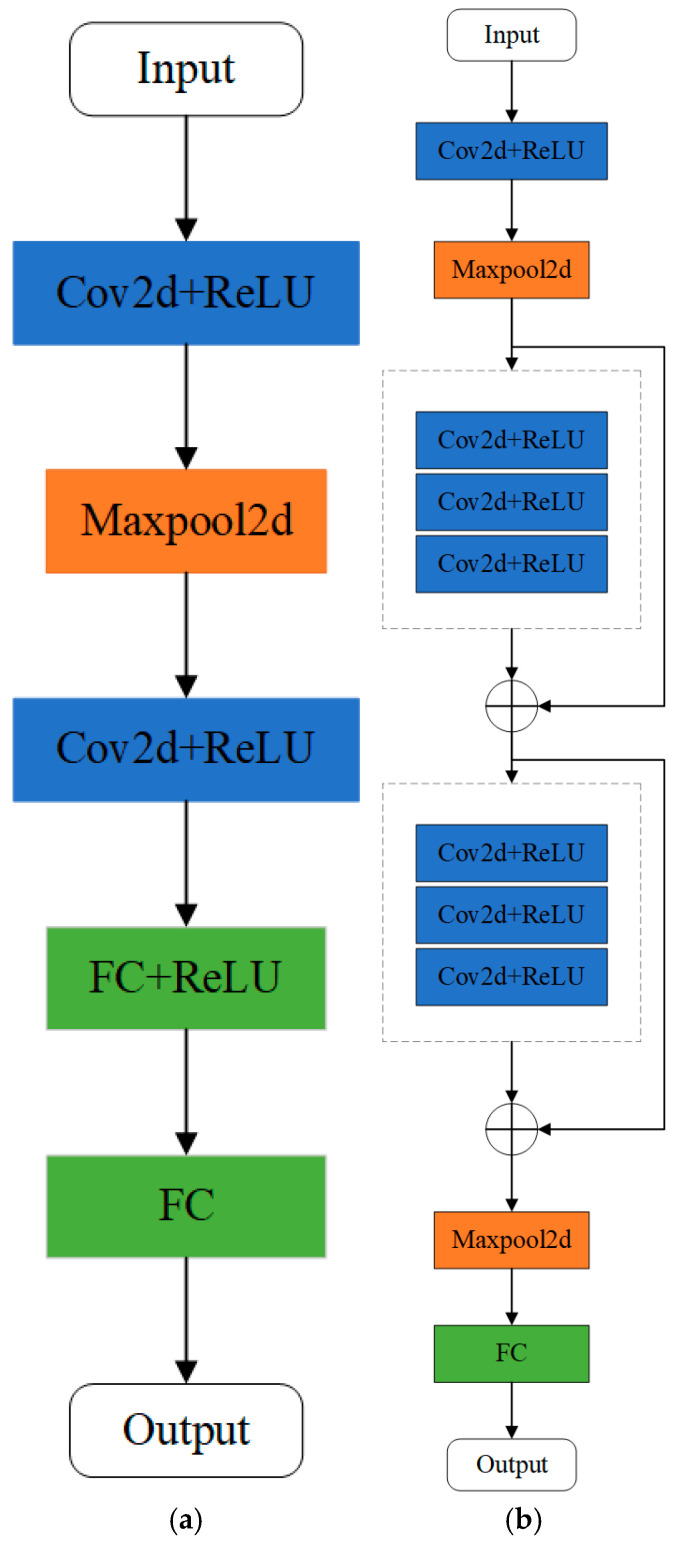
CNN and ResNet network architecture diagram. (**a**) CNN network; (**b**) ResNet network.

To verify the effectiveness of the proposed federated edge-smart approach in this paper, as shown in Figure 8, in this study, the NVIDIA Jetson Nano (Hunan Chuanglebo Intelligent Technology Co., Ltd., Changsha, China) is adopted as the edge intelligent device for model deployment. A heterogeneous platform is simulated using three edge intelligent devices equipped with different computing power resources, and their detailed information is presented in Table 1. Programming was conducted using PyCharm 2024, with Python 3.9 as the programming language, pytorch version 1.12.0, and CUDA version 12.6.

In this study, two heterogeneous models, CNN and ResNet, are randomly deployed to each client for training on the EuroSAT and RSSCN7 datasets. The network architectures of CNN and ResNet are shown in Figure 8, respectively. The learning rate (lr) for federated learning is set to 0.01. Both the global communication rounds and the local training rounds for each client are set to 500. The number of clients is configured to be 10, the data-sharing ratio is set to 0.2, and the aggregation threshold is set to 1.0. The subsequent ablation experiments and comparative experiments were each run independently 20 times, and the average values of the experimental metrics over these 20 runs are presented in the tables.

### 4.3. Ablation Experiment

To assess the independent contributions of the Data Sharing Mechanism (DSM) and the Intelligent Model Aggregation Strategy (IMAS) to model performance, this paper designs a systematic ablation study. The experimental results, as presented in Table 2, provide a quantitative analysis of the impact of each module on model performance.

The experimental results demonstrate that the introduction of the data sharing mechanism improved F1 scores by 0.09 on the FEMNIST dataset and 0.17 on the FEAIR dataset, and improved the F1 score by 0.24 on the EuroSAT dataset and by 0.18 on the RSSCN7 dataset. These findings significantly validate the effectiveness of the data sharing strategy in mitigating the Non-IID (non-independent and identically distributed) characteristics of the data. By constructing a shared dataset, the strategy effectively alleviates the data distribution discrepancies among edge platforms.

Furthermore, the incorporation of the intelligent model aggregation strategy reduced the gradient difference by 2.1403 on the FEMNIST dataset, 2.1146 on the FEAIR dataset, 1.0483 on the EuroSAT dataset, and 2.3264 on the RSSCN7 dataset; it also significantly decreased the number of communication rounds required for model convergence. These results demonstrate that the similarity-based model aggregation strategy can effectively mitigate the negative impacts of device heterogeneity. By filtering high-quality local model updates, it reduces the model’s consumption of communication resources and enhances the stability and convergence of the global model.

### 4.4. Comparative Experiment

Table 3 presents the experimental results of FedAvg, FedProx, FedCosA, FedBN, FedMAE, and the proposed DSIA-FEI algorithm on the FEMNIST, FEAIR, EuroSAT, and RSSCN7 datasets. Compared to FedAvg, FedProx, FedCosA, FedBN, and FedMAE, the DSIA-FEI algorithm achieves higher overall prediction accuracy and F1 scores. It also reduces parameter differences among local models on edge platforms and decreases communication rounds for convergence.

In 20 independent experimental runs, the DSIA-FEI algorithm demonstrated strong stability. On all datasets, its accuracy and F1 score values ranged from 0.85 to 0.95, with variances of 0.34 and 0.15, respectively. The gradient discrepancy values were between 0.2 and 0.5, with variances of 0.08 and 0.13. These results confirm the stability of the DSIA-FEI algorithm.

**Figure 9:**
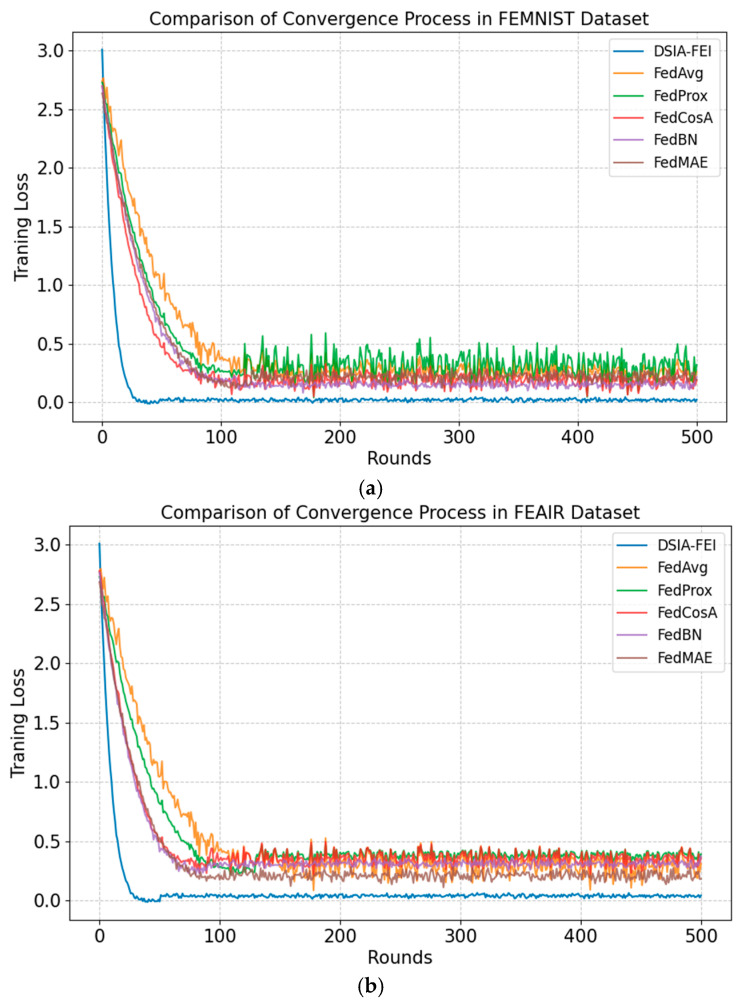

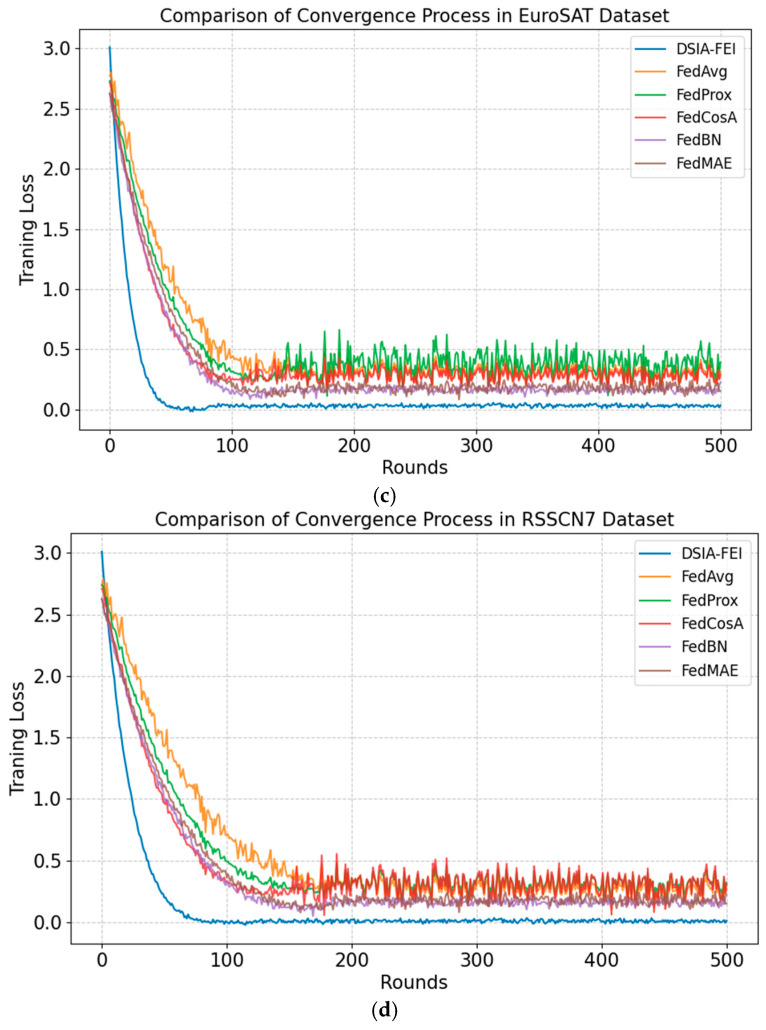
Convergent curves on all training sets. (**a**) FEMNIST training set; (**b**) FEAIR training set; (**c**) EuroSAT training set; (**d**) RSSCN7 training set.

Figure 9 illustrates the convergence processes of DSIA-FEI, FedAvg, FedProx, FedCosA, FedBN, and FedMAE on the training sets of FEMNIST, FEAIRm, EuroSAT, and RSSCN7. DSIA-FEI not only outperforms the other baseline algorithms in terms of convergence speed and stability but also demonstrates superior overall performance. This is attributed to the intelligent model aggregation strategy introduced in DSIA-FEI, which optimizes the global model update process by reducing unnecessary parameter updates. As a result, DSIA-FEI accelerates overall convergence and enhances the model’s learning capability across the datasets.

## 5. Conclusions

Aiming at the problems of multi-source heterogeneous data and heterogeneous devices and models in cooperative data processing of unmanned platforms, this paper proposes a cooperative data processing method for unmanned platforms based on federated edge intelligence, in which the remote sensing image dataset is processed by simulating the Non-IID characteristics. The simulation experiments are carried out on the dataset with inherent Non-IID characteristics and the constructed simulated Non-IID characteristics; the effectiveness of the data sharing strategy and the intelligent model aggregation strategy based on similarity measurement and loss gradient are verified and compared with the existing baseline methods. It is verified that the proposed method has high accuracy and stability for data processing which are obviously better than the existing methods. In addition, this method can significantly alleviate the impact of heterogeneous data, devices, and models on data processing, and reduce the consumption of communication resources of resource-constrained devices such as unmanned platforms.

## 6. Future Perspectives

Heterogeneity in data, devices, and models represents a pivotal challenge for unmanned clusters during collaborative data processing. While the federated edge intelligence approach proposed in this paper can effectively alleviate this issue, further practical and complex applications of unmanned swarms are bound to expose more technological bottlenecks and key research questions. Based on this study, the following directions can be pursued for future research and expansion:

(1) Privacy Protection Strategies: The data sharing strategy employed in this paper can effectively alleviate data heterogeneity. However, there are still data leakage and privacy risks during data sharing between edge and end-device unmanned platforms. In the future, we need to further explore privacy/utility trade-off mechanisms for data sharing in open environments. For example, we can organically integrate secure enhancing technologies such as differential privacy and homomorphic encryption with the federated learning framework. In addition, constructing verifiable privacy protection paradigms will be crucial.
(2) Cross-Modal Data Fusion: The federated edge intelligence framework in this paper solves the non-IID property of heterogeneous data, but has not fully considered the semantic correlation of cross-modal data. As unmanned swarms may collect and process multi-modal data in practical scenarios, future work can explore cross-modal federated learning frameworks. By using knowledge distillation and building hierarchical semantic alignment networks, deep feature fusion of multi-modal data like images, sounds, and electric currents can be achieved.
(3) Hybrid Simulation System Construction: The experimental verification of the federated edge intelligence method proposed in this paper is mainly based on simulation and limited real data, lacking adaptive verification in complex dynamic scenarios. With the rapid development of hybrid reality and digital twin technologies, future research can design hybrid simulation systems with multi-physics coupling. Creating high-fidelity unmanned swarm training environments to simulate complex scenarios like battlefields and urban canyons can validate the algorithm’s robustness in diverse conditions.

## Figures and Tables

**Table 1:** Information on different types of NVIDIA Jetson Nano edge-smart devices.

	CPU	GPU	Memory
Jetson Nano Developer Kit	Quad-core Arm Cortex-A57 MPCore	128-core NVIDIA Maxwell architecture GPU	4 GB 64-bit LPDDR4
Jetson Orin Nano 4 GB	6-core Arm Cortex-A78AE v8.2	512-core NVIDIA Ampere architecture GPU	4 GB 64-bit LPDDR5
Jetson Orin Nano 8 GB	6-core Arm Cortex-A78AE v8.2	1024-core NVIDIA Ampere architecture GPU	8 GB 128-bit LPDDR5

**Table 2:** Ablation study on the datasets.

Dataset	Methods	Accuracy	F1_score	${Grad}_{diff}$	Rounds
FEMNIST	FedAvg	0.7489	0.73	2.6874	144
	w/o DSM	0.8142	0.82	1.3658	112
	w/o IMAS	0.7774	0.79	0.5471	71
FLAIR	FedAvg	0.7632	0.69	2.3987	150
	w/o DSM	0.8346	0.86	1.2471	74
	w/o IMAS	0.7936	0.81	0.2841	60
EuroSAT	FedAvg	0.7702	0.61	2.0697	102
	w/o DSM	0.8207	0.85	1.3698	96
	w/o IMAS	0.8390	0.83	1.0214	82
RSSCN7	FedAvg	0.7495	0.70	2.6471	220
	w/o DSM	0.8109	0.88	1.2684	183
	w/o IMAS	0.7943	0.84	0.3207	150

**Table 3:** Comparative experiment on the datasets.

Dataset	Methods	Accuracy	F1_score	${Grad}_{diff}$	Rounds
FEMNIST	FedAvg	0.7489	0.73	2.6874	144
	FedProx	0.8017	0.79	2.3014	120
	FedCosA	0.8124	0.79	0.6541	95
	FedBN	0.8236	0.78	2.0674	114
	FedMAE	0.8147	0.80	1.9874	126
	DSIA-FEI	0.9087	0.90	0.1789	48
FLAIR	FedAvg	0.7632	0.69	2.3987	150
	FedProx	0.8336	0.82	1.3654	130
	FedCosA	0.8324	0.79	2.0347	89
	FedBN	0.8321	0.80	2.9874	90
	FedMAE	0.7961	0.81	2.4789	103
	DSIA-FEI	0.9130	0.88	0.3654	51
EuroSAT	FedAvg	0.7702	0.61	2.0697	152
	FedProx	0.7804	0.78	2.0314	141
	FedCosA	0.8265	0.82	1.3654	115
	FedBN	0.7813	0.80	2.3654	132
	FedMAE	0.8126	0.81	2.2745	145
	DSIA-FEI	0.8792	0.86	0.4127	80
RSSCN7	FedAvg	0.7495	0.70	2.6471	220
	FedProx	0.7980	0.88	1.6874	183
	FedCosA	0.8147	0.85	1.8415	151
	FedBN	0.7869	0.80	3.2471	177
	FedMAE	0.8062	0.86	2.6815	187
	DSIA-FEI	0.8656	0.90	0.2314	122

## Data Availability

The FEMNIST dataset in this paper is available at https://leaf.cmu.edu (accessed on 6 March 2025). The FEAIR dataset in this paper is available at https://github.com/apple/ml-flair (accessed on 6 March 2025). The EuroSAT dataset in this paper is available at https://github.com/phelber/eurosat (accessed on 6 March 2025). The RSSCN7 dataset in this paper is available at https://aistudio.baidu.com/datasetdetail/52117 (accessed on 6 March 2025).

## Abbreviations

The following abbreviations are used in this manuscript:

Non-IID	Non-Independent and Identically Distributed
DSM	Data Sharing Mechanism
IMAS	Intelligent Model Aggregation Strategy
FedAvg	Federated Averaging
FedProx	Federated Proximal
FedCosA	Federated Using Cosine Annealing
FEI	Federated Edge Intelligence
DSIA-FEI	Federated Edge Intelligence Based on Data Sharing and Intelligent Model Aggregation
